# 

**Published:** 1837

**Authors:** 


### BIBLIOGRAPHICAL RECORD;

### OR QUARTERLY LIST OF NEW PUBLICATIONS IN MEDICINE AND SURGERY, AND THEIR ASSOCIATE SCIENCES.

---

### American.

1. A Manual of the Diseases of the Eye. By S. Littell, Jr., M. D. One of the Surgeons of the Wills' Hospital for the Blind and Lame. 12mo. pp. 255. Philadelphia: 1837.

2. Principles of Pathology and Practice of Physic. By John Mackintosh, M. D., Lecturer on the Practice of Physic in Edinburgh. Second American from the fourth London edition; with Notes and Additions, by Samuel G. Morton, M. D., late Physician to the Philadelphia Alms-House Hospital, and Lecturer on Pathological Anatomy. 2 vols. 8vo. pp. 563 and 568. Philadelphia: 1837.

3. Plates of the Cerebro-Spinal Nerves, with references, for the use of Medical Students. By Paul B. Goddard, M. D., Prosector of Anatomy in the University of Pennsylvania, Member of the Academy of Natural Sciences, &c. 4to. pp. 60. Philadelphia: 1837. —*See last No.*

4. A Treatise on Functional and Organic Diseases of the Uterus; from the French of T. Duparcque, M. D. Paris. Translated, with Notes, by Joseph Warrington, M. D. 8vo. pp. 455. Philadelphia: 1837.—*See review in the present No.*

5. Illustrations of Pulmonary Consumption; its anatomical characters, causes, symptoms, and treatment; to which are added some remarks on the Climate of the United States, the West Indies, &c. with thirteen plates, drawn and colored from nature. Second Edition. By S. G. Morton, M. D., late Physician to the Alms-House Infirmary. 8vo. pp. 349. Philadelphia: 1837.

6. An Examination of Phrenology, in two Lectures, delivered to the Students of Columbia College, February, 1837. By Thomas Sewall, M. D., Professor of Anatomy and Physiology. With plates, 8vo. Washington City: 1837.

7. A Treatise on the Malformations, Injuries and Diseases of the Rectum and Anus, illustrated with nine 4to. plates. By George Bushe, M. D., formerly Professor of Anatomy and Physiology, &c. 8vo. pp. 299. New York: French & Allard. 1837.—*To be more particularly noticed in our next.*

8. A Dictionary of Practical Medicine. By James Copland, M. D. Part iii. Boston: W. D. Ticknor. 1837.

9. Address delivered before the Medical Society of the State of New York, February 8, 1837. By James McNaughton, M. D. President of the Society. Albany: 1837.

10. Oration on the Guidance of a Sound Philosophical Spirit in the Investigation of Medical Science,

Read before the Cincinnati Medical Society, January 4th, 1837. By John P. Harrison, M. D., Professor of Materia Medica in the Medical Department of the Cincinnati College. Printed by order of the Society. Cincinnati: 1837. —*Noticed in this No.*

11. Surgical Observations on Tumors; with numerous colored engravings. By John C. Warren, M. D., Professor of Anatomy in Harvard University. 8vo. pp. 600. Boston: 1837.—*To be reviewed in our next.*

12. Homœopathic Medicine, illustrating its Superiority over the other Medical Doctrines, with an account of the Regimen to be followed during the Treatment of Disease. By M. Croserio, M. D., President of the Homœopathic Society of Paris, &c. &c. Translated from the French, with Notes, by C. Neidhard, M. D. 12mo. pp. 89. Philadelphia: 1837—.*See review department.*

BRITISH.

1. The Human Brain, its Configuration, Structure, Development and Physiology; illustrated by references to the Nervous System in the lower orders of Animals. By Samuel Solly, Lecturer on Anatomy and Physiology in St. Thomas' Hospital. 8vo. pp. 492, with 12 plates. London: 1836.

2. A Treatise on Tetanus; being the Essay for which the Jacksonian Prize for the year 1834, was awarded by the Royal College of Surgeons in London. By T. B. Curling, Assistant Surgeon to the London Hospital. 8vo. pp. 236. London: 1836.

*This erudite little work is republished in Professor Dunglison's excellent "Select Medical Library."*

3. Experimental Researches into the Physiology of the Human Voice. By John Bishop, M. R. C. S. pp. 24, with plates. London: 1836.

4. A New and Familiar Treatise on the Structure of the Ear and on Deafness. By A. W. Webster, M.D. 8vo. pp. 151. London: 1836.

5. Elements of Medicine—Vol. 1. on Morbid Poisons. By Robert Williams, M. D., Senior Physician to St. Thomas' Hospital—8vo. pp. 342. London: 1836.

5. Elements of the Practice of Physic; presenting a view of the present state of Special Pathology and Therapeutics. By David Cragie, M. D. F. R. S. E., Physician to the Royal Infirmary, Edinburgh, &c. &c.—vol. 1. 8vo. pp. 952. Edinburgh: 1836.

*Dr. Cragie's work, which comprehends an account of the diseases usually included under the heads of fevers, cutaneous inflammations, and mucous inflammations, is decidedly one of the most elaborate and erudite productions of the age.*

6. Elements of the Practice of Medicine. By Richard Bright, M. D. and Thomas Addison, M. D., Lecturers on the Practice of Medicine at Guy's Hospital. Part i.—8vo. pp. 129. London: 1836.

7. Elementary System of Physiology—complete in one volume. By John Bostock, M. D. F. R. S. &c. 3d Edition, revised and corrected throughout—8vo. pp. 887. London: 1836.

8. The Principles and Practice of Obstetric Medicine, in a series of Systematic Dissertations on Midwifery and on the Diseases of Women and Children. Illustrated by numerous plates. By D. D. Davis, M. D. M. R. S. L., Professor of Midwifery in the University of London, &c.—8vo. pp. 1294. London: 1836.

9. A Practical Treatise on the Management and Diseases of Children. By K. T. Evanson, M. D., Professor of Medicine, and H. Maunsell, M. D., Professor of Midwifery in the Royal College of Surgeons in Ireland—8vo. pp. 527. Dublin: 1836.

10. The Principles of Surgery. By James Syme, F. R. S. E., Professor of Clinical Surgery in the University of Edinburgh.—8vo. pp. 460. Edinburgh: 1836.

11. Outlines of a Course of Lectures on Medical Jurisprudence. By T. S. Traill, M. D., Professor of Medical Jurisprudence in the University of Edinburgh, &c. &c. Small 8vo. pp. 94. Edinburgh: 1836.

12. Clinique Medicale ; or Reports of Medical Cases. By G. Andral, M. D., &c. &c. Translated by D. Spillan, M. D. Part v. 8vo. London: 1836.

*This is the concluding number of this valuable work.*

13. A Medical Vocabulary; or, Explanation of all Names, Synonymes, Terms, and Phrases used in Medicine and Surgery, &c. By a Medical Practitioner.—12mo. pp. 185. Edinburgh: 1836.

14. A Bedside Manual of Physical Diagnosis, applied to Diseases of the Lungs, Pleura, Heart, Vessels, Abdominal Viscera, and Uterus. By Charles Cowan, M. D., P. & E., &c. 18mo. pp. 58. London: 1836.

15. A Practical Demonstation of the Human Skeleton. By George Elkington, M. R. C. S., Demonstrator of Anatomy in the Royal School of Medicine and Surgery, Birmingham. 12mo. pp. 240.—London and Birmingham: 1836.

16. Lectures on the Morbid Anatomy of the Serous and Mucous Membranes. In two volumes. By Tho's Hodgkin, M. D., Demonstrator of Morbid Anatomy, and Curator of the Museum at Guy's Hospital, &c. &c. 8vo. London: 1836.

17. An Essay on the Origin and Nature of Tuberculous and Cancerous Diseases. Read to the Medical Section of the British Association. By Richard Carmichael, M. R. T. A., Consulting Surgeon of the Richmond Surgical Hospital. 8vo. pp. 56. Dublin: 1836.

18. A Description of the Bones, together with their several connections with each other, and with the muscles. Specially adapted for students in Anatomy. By J. F. South, Assistant-Surgeon to St. Thomas' Hospital. Third edition, with 250 Engravings.—London: 1836.

19. The Retrospective Address upon Medical Science and Literature, delivered at the Fourth Anniversary Meeting of the Provincial Medical and Surgical Association, held at Manchester, July 21, 1836. By John Green Crosse, Esq., F. R. S., Surgeon to the Norfolk and Norwich Hospital. 8vo. pp. 88. Worcester: 1836.

20. Practical Observations on various subjects relating to Midwifery. By James Hamilton, M. D., F. R. S., E. Professor of Medicine and Midwifery, &c., in the University of Edinburgh. Part I. 8vo. pp. 317. Edinburgh: 1836.

21. Lectures on the Nervous System, and its Diseases. By Marshall Hall, M. D. 8vo. pp. 171. London: 1836.

22. Elements of Medical Jurisprudence. By Alfred S. Taylor, F. R. S., Lecturer on Med. Juris. and Chemistry in Guy's Hospital. Vol I. 8vo. pp. 511. London: 1836.

FRENCH.

1. Texture et Developement des Poumons. These soustenue, 24 Juin, 1836. Par M. A. Berard, Chirurgien de l'Hôpital Necker. 8vo. pp. 132. Paris: 1836.

2. Tràite Pratique de la Syphilis. Par le Baron Phillippe Boyer. 8vo. pp. 284. Paris: 1836.

3. Le Système Lymphatique, considérée sous les Rapports Anatomique, Physiologique, et Pathologique. Par G. Breschet, M. D., &c. 8vo. pp. 304. IV. Planches. Paris: 1836.

4. Mémoire sur les Rapports des Sexes dans les Naissances de l'Espéce Humaine. Par Ch. Girou de Buzareingues. 8vo. Paris: 1836.

5. De la Prostitution dans la Ville de Paris, considérée sous la Rapport de l'Hygiène Publique, de la Morale, et de l'Administration. Par A. T. B. Parent-Duchatelet, M. D., &c. 2 vols. 8vo. Paris : 1836.

6. Médicine Légale, Theorique et Pratique. Par Alph. Devergie, M. D., Professeur aggrégé de la Faculté de Médicine de Paris, Professeur de Médicine Légale et de Chèmie Médicale, &c. &c. Tomes I—II. 8vo. pp. 724—422. Paris : 1836.

GERMAN.

1. Lehrbuch der Geburtshülfe fur Hebammen. Von F. R. Nägele, M. D., Professor der Medizin und Geburtshülfe zu Heidelberg, &c. Dritte Auflage. 8vo. pp. 406. Heidelberg : 1836.

2. Katechismus der Hebammen Kunst, als Anhang zu Seinem Lehrbuche, &c. Von Dr. F. K. Nägele. Dritte Auflage. 8vo. pp. 127. Heidelberg: 1836.

3. Beobachtungen aus dem gebiete der Pathologie und Pathologischen Anatomie, gesammelt von Dr. T. F. H. Albers, Professor der Medizin on der Universität zu Bonn. Erster Theil. 8vo. pp. 204. Bonn : 1836.

4. Commentatio de Delirio Tremente. Auctore O. C. Hoegh-Gulberg, M. D. 8vo. pp. 293. Hauniæ; 1836.

5. De Blessahooplastice, Diss. Inaug. Med. Auctore E. O. Peters. 4to. pp. 43. Leipsiæ : 1836.

6. Dissertatio de Methodo Endermatica. Auctore O. Abremsen., M. D. 8vo. pp. 267. Hauniæ: 1836.

7. De Febre Puerperali Maligna. Auctore S. T. Ballina. 8vo. pp. 94. Hauniæ : 1836.

8. De Fungo Articulari, Dissertatio. Auctore Michaele Djoress. 8vo. pp. 116. Hauniæ : 1836.

9. Tractatus de Connexu inter Nervum Vagum and Accessorium Willisii. Auctore H. C. B. Bendz. 4to. pp. 60. Hauniæ : 1836.

10. Uber die Endungsweise der Nerven in den Muskeln, nach einigen Untersuchungen. Von Fr. C. Emmert, Privat-Docent an der Hochschule in Bern. Mit 2 Abbildungen. 4to. pp. 35. Bern : 1836.

11. Specimen Historico-Medicum de Choleræ Asiaticæ itinere per Belgium Septentrionale. Auctore A. C. G. Suerman, M. D. 8vo. pp. 289. Trajecti ad Rhenum: 1836.

12. Denkwürtigkeiten in der Artzlichen Praxis. Von Dr. T. H. Kopp. In 3 bender. Frankfurt : 1836.

13. Handbuch der Menschlichen Anatomie. Von C. F. H. Krause, M. D., Professor der Anatomie, &c., zu Hannover. 8vo. I. II. Abtheilung. Hannover : 1836.

14. Supplemente zur Lehre vom Kreislaufe. II. Heft. Flèmmerbewegungen, leben Blutsphären, und Monaden lehre. Von Dr. A. F. J. C. Meyer, Ord. Off. Professor der Anatomie und Physiologie zu Bonn. 4to. pp. 55. Bonn : 1836.

15. Die Erkenntniss und Heilung der Ohrenkrankheiten. Von Dr. Wilhelm Kramer. Mit Kupfertaflen. 8vo. pp. 400. Berlin : 1836.

16. Uber die Harnblasenspaltè, nebst Beschreibung und Abbildung einiger beim Menlichen und Weiblichen Geschlechte Beobachten fællen. Von Peter Schmitt, M. D. &c. 4to. pp. Wurtzburg : 1836.

